# Environmental factors modulate β-lactamase phenotypes: effect of pH and redox potential on β-lactam resistance

**DOI:** 10.1128/spectrum.01453-26

**Published:** 2026-07-17

**Authors:** Mikkel Anbo, Jacob Dyring Jensen, Mirena Ivanova, Hanne Nørgaard Nielsen, Christina Aaby Svendsen, Saria Otani, Frank M. Aarestrup

**Affiliations:** 1 DTU National Food Institute, Technical University of Denmark 5205, Kgs-Lyngby, Denmark; JMI Laboratories, North Liberty, Iowa, USA

**Keywords:** anaerobiosis, pH, susceptibility, β-lactamases, β-lactams

## Abstract

Environmental factors may have unnoticed effects on the activity of β-lactamases against β-lactams. We constructed a panel of *Escherichia coli* strains expressing 16 different β-lactamases and determined the MICs of 10 β-lactams under aerobic conditions at different pH values and under anaerobic conditions. We observed a contrasting effect of pH on MICs depending on the β-lactam and β-lactamase. Anaerobiosis generally increased MICs, but the effect depended completely on the exact gene and β-lactam.

**IMPORTANCE:**

Huge efforts have gone into standardization of antimicrobial susceptibility testing. However, bacteria do not live under standardized conditions and the possible importance of constantly changing environmental conditions on the efficacy of antibiotics and resistance genes might have been overlooked. Here we assess the influence of media pH and anaerobiosis on the resistance (MIC) of 16 widespread and critical β-lactamases. We observed a contrasting effect of pH on MICs depending on the β-lactam and β-lactamase, while anaerobiosis generally increased MICs, but with effects depending on the exact gene and beta-lactam. This can have major importance for our understanding of interpretation of standardized in vitro MIC results for treatment, as well as ecology and evolution of AMR.

## OBSERVATION

Antimicrobial resistance is a growing health threat, resulting in huge morbidity and mortality in humans and animals, as well as great economic losses (1). The most important factor for increased AMR is widespread antimicrobial usage to treat human infections, to treat and prevent infections in livestock, as well as selection by antibiotics excreted into the environment, even though several other epidemiological factors also contribute (2).

Globally, most phenotypic AMR data come from susceptibility testing performed under strictly standardized conditions as part of routine clinical diagnostics to guide treatment in humans. However, it has been known for a long time that the activity of antibiotics depends on the exact micro-environmental conditions, as we and others recently showed also for acquired resistance (3, 4).

Ortiz-Miravalles et al. (3) showed a profound effect of anaerobiosis on 136 different resistance genes against eight antibiotic families, while Anbo et al. (4) showed effects of pH and temperature on two β-lactamases (*bla*_CTX-M-15_ and *bla*_CMY-2_), including verification of this being related to a direct effect on the enzymatic reaction. These genes belong to Ambler classes A and C, respectively, and both confer resistance to third-generation cephalosporins, but not carbapenems (5). The study by Anbo et al. (4) established that environmental conditions can modify acquired resistance phenotypes, but it remains unclear whether such effects are generalizable across β-lactamase families, β-lactam substrates, and oxygen conditions. In particular, previous mechanistic work focused on a limited number of enzymes, whereas the extent to which pH and anaerobiosis reshape resistance across a broader β-lactamase panel remains unresolved.

β-Lactamases are divided into four Ambler classes: A, B, C, and D, where classes A, C, and D are serine β-lactamases, and class B accommodates one or two zinc ions at the active sites and are classified as metallo-β-lactamases, which are further divided into three groups: B1, B2, and B3 (5, 6). Especially, metallo-β-lactamases and other β-lactamases conferring resistance to carbapenems are considered a major global health threat (7).

To assess whether our previous results, which investigated the influence of medium pH on the MIC of two β-lactamases, are indicative of a more general phenomenon, we constructed a panel of 16 isogenic *Escherichia coli* K12 strains, each expressing different β-lactamases and covering classes A, B1, and D, as well as small-spectrum β-lactamases and cephalosporinases, but with a specific focus on carbapenemases and clinical relevance. All engineered strains were generated in the same *E. coli* MG1655 chromosomal background and therefore retained the native chromosomal *ampC* gene; no *ampC* deletion was introduced. The isogenic strains were tested aerobically at four pH levels (pH 5, 6, 7, and 8) and under anaerobic conditions (pH 7) to 10 different β-lactams and β-lactamase-inhibitor combinations (with or without 4 µg/mL clavulanic acid). Anaerobiosis susceptibility testing was performed in an anaerobic chamber using pre-reduced media and bacteria grown anaerobically. Cloning, growth conditions, susceptibility testing, and data analyses were conducted as described by Anbo et al. (4), and the strains, plasmids, and primers used are given in Tables S1 and S2.

### Effects of medium pH are gene- and substrate-specific

**Fig 1:**
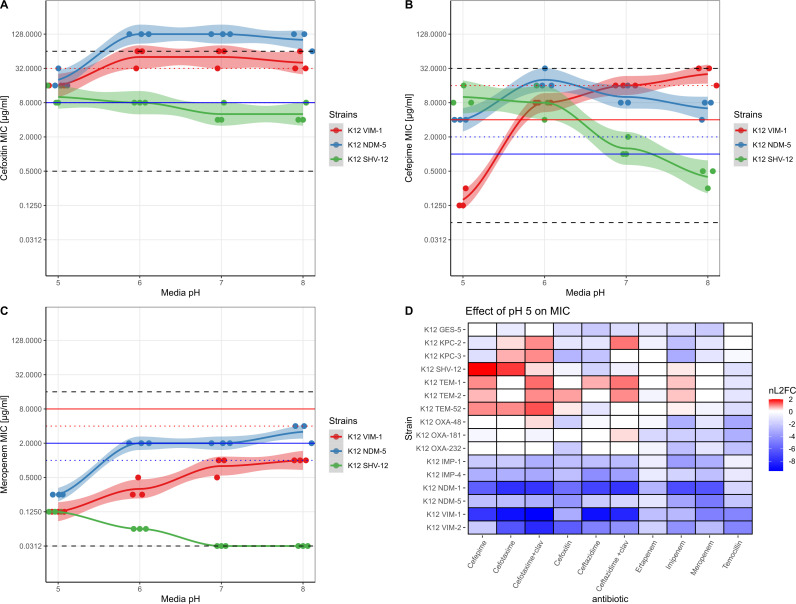
Influence of pH on the MIC of different β-lactamases.** (A–C)** Effect of medium pH on the MIC of a subset of β-lactamases to selected β-lactams. Each line shows the smoothed conditional mean, and the three similar colored points represent the MIC for each test. Each β-lactamase shows a distinct pH optimum, suggesting that pH can play an integral role in shaping resistomes. Dashed lines indicate the maximum and minimum concentrations that were assayed, while the horizontal red and blue lines indicate resistant and susceptible breakpoints in Enterobacterales (EUCAST breakpoints v16), respectively. Dotted horizontal blue and red lines indicate CLSI susceptibility and resistance breakpoints, respectively. (**D)** Heatmap showing the effect of media at pH 5 on MIC, where each substrate/gene combination is colored according to the log2 fold change between the MIC at pH 5 and the MIC at pH 7 (red for increased resistance and blue for increased susceptibility). This was normalized to the corresponding log2 fold change in MIC of the wild type (Fig. S3(1)), to provide a parental-background-corrected estimate. Each condition was tested with three replicates. β-Lactamases are organized according to Ambler class. Additional data are provided in Fig. S1.

For most substrate/gene combinations, we found that the MIC varied by at least twofold across the pH range that was assayed (Fig. 1 and Fig. S1 and S3); however, some strains varied in their MIC by at least 512-fold. Thus, the MIC of K12 VIM-1 to cefotaxime + clavulanic acid exceeded the highest concentration in the panel at pH ≥ 7 but was susceptible to most cephalosporins at pH 5. In some cases, the changes in MIC at low (pH 5) versus high (pH 8) pH crossed the breakpoints for susceptibility and resistance, as established by EUCAST/CLSI (Fig. 1 and Fig. S1). This, however, also varied by substrate/gene combinations. Thus, for example, for temocillin, the MIC values for most gene constructs went from higher to lower, while for cefotaxime and ceftazidime, it was from lower to higher, and for cefepime, both patterns were observed. Large variations were observed; however, some correlations between genes of the same Ambler class might be present, with all metallo-β-lactamases showing increased susceptibility at pH 5 for all β-lactams, whereas the opposite was found for some class A β-lactamases and selected β-lactams. As a control, we tested the wild-type K12 MG1655, which also expresses an innate β-lactamase AmpC. Because all constructed strains were generated in this *E. coli* MG1655 background, both the parental control and the β-lactamase-expressing derivatives retained the native chromosomal *ampC* gene. The parental strain was susceptible to most treatments; however, low pH (pH 5) increased the MIC by 2–16-fold depending on the substrate, especially for imipenem. We therefore normalized environmentally induced MIC changes in each β-lactamase-expressing strain to the corresponding MIC change in the parental K12 strain. This normalization controls for environmental effects shared by the K12 background, including intrinsic susceptibility, growth effects, substrate stability, and any contribution from native AmpC. However, because an *ampC* deletion strain was not included, the normalized values should be interpreted as parental-background-corrected MIC shifts associated with the introduced β-lactamase, rather than as complete biochemical isolation of the introduced enzyme alone.

### Low oxygen increases resistance for most β-lactamases

**Fig 2:**
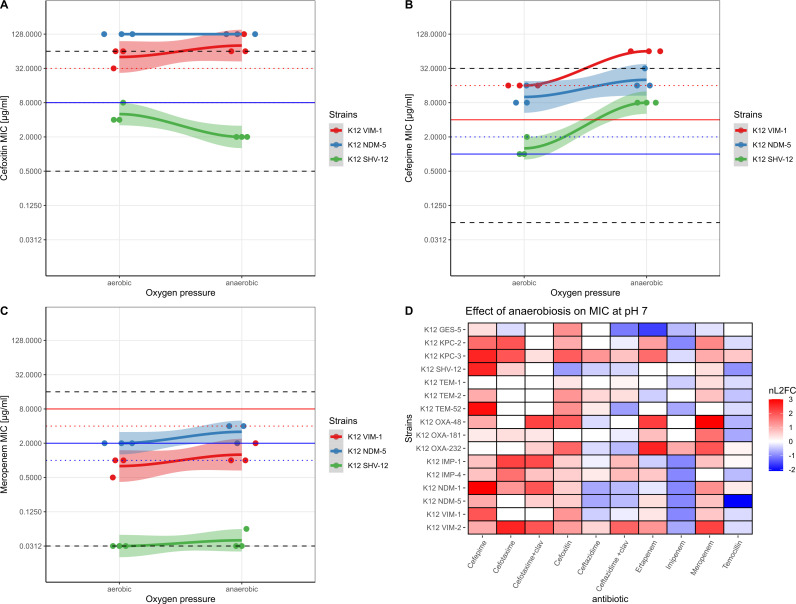
Influence of oxygen pressure on the MIC of different β-lactamases at pH 7. (**A–C)** Effect of anaerobiosis on the MIC of a subset of β-lactamases to selected β-lactams. Each line shows the smoothed conditional mean, and the three similarly colored points represent the MIC for each test. Dashed lines indicate the maximum and minimum concentrations that were assayed, while the horizontal red and blue lines indicate resistant and susceptible breakpoints in Enterobacterales (EUCAST breakpoints v16), respectively. Dotted horizontal blue and red lines indicate CLSI susceptibility and resistance breakpoints, respectively. (**D)** Heatmap showing the effect of anaerobiosis on MIC, where each substrate/gene combination is colored according to the log2 fold change between the anaerobic MIC and the aerobic MIC (red for increased resistance and blue for increased susceptibility). This was normalized to the corresponding log2 fold change in MIC of the wild type (Fig. S3(2)), to provide a parental-background-corrected estimate. Each condition was tested with three replicates. β-Lactamases are organized according to Ambler class. Additional data are provided in Fig. S2.

Similar to pH, there exist large oxygen gradients in the human body, going from highly oxygenated tissue to anaerobic conditions. One example of this is in the gastrointestinal tract, where oxygen is rapidly consumed and obligate anaerobic bacteria can proliferate. Overall, we observed an increase in the MIC for most gene/substrate combinations under anaerobic conditions, with 2–16-fold increases in the MIC (Fig. 2 and Fig. S2 and S3(4)), and in a few cases where the isolates would change from being classified as susceptible to resistant using current EUCAST/CLSI breakpoints. However, for imipenem and temocillin, we found completely opposite effects. For cefepime, we observed an increase in MIC for almost all strains (except three), which suggests that the enzymatic breakdown of this substrate is especially sensitive to oxygen pressure. No obvious correlation between patterns and Ambler class could be observed, and also of note is that even for very closely related β-lactamases, such as the different blaOXA genes (OXA-48, OXA-181, and OXA-232), we found much larger jumps in the ertapenem MIC of OXA-48 (4.8-fold) and OXA-232 (sixfold) when cultured anaerobically, compared to OXA-181 (1.7-fold). OXA-181 only differs from OXA-232 by one amino acid substitution, S214R. On the other hand, OXA-48 differs by four AA substitutions from OXA-181, with arginine at position 214. This could suggest that OXA-181 has evolved to be more stable in an oxygen-rich environment compared to similar variants.

It was observed already 40–50 years ago that exact environmental growth conditions could drastically influence the susceptibility of bacteria to various antimicrobial agents. More recently, we and others have observed that this is also the case for bacteria with acquired resistance (3, 4, 8–14), possibly due to the specific influence of enzymatic reactions (4).

Redox potential and pH play a large role in modulating the folding, activity, and stability of proteins excreted across the inner membrane (especially those that are exported extracellularly). Past experiments have shown that the pH of the *E. coli* periplasmic space is equal to the extracellular environment, with direct effects on periplasmic proteins (15), such as β-lactamases. It is well established that pH affects not only enzyme activity but also folding and stability, and that the dissolved oxygen content also directly affects extracellular and periplasmic proteins, with effects on function and stability (16).

We observed that all metallo-β-lactamases showed increased susceptibility at pH 5. It has previously been shown that the metallo-β-lactamase βLII from *Bacillus cereus* is reversibly inactivated at low pH, due to dissociation of the two zinc(II) equivalents (17). Whether this is a generalizable phenomenon for all metallo-β-lactamases and whether this can have implications for clinical treatment would, however, require further investigations.

In general, we observed an increase in the resistance of most β-lactamases during anaerobiosis, and we mainly attribute this to the more reducing anaerobic environment that, relatively, supports the stability of β-lactamases compared to the oxidizing aerobic environment. However, the effect depended completely on the exact gene and β-lactam, and even for closely related compounds such as imipenem and meropenem, the effect of anaerobiosis was completely opposite.

A limitation of this study is that anaerobiosis was tested only at pH 7, whereas pH and oxygen tension can vary simultaneously *in vivo*. We chose to examine pH and anaerobiosis as separate environmental axes to allow systematic comparison across a broad panel of 16 β-lactamases and 10 β-lactam compounds. Importantly, the novelty of this study is to show the strong gene- and substrate-specific effects, which suggest that the combined effect of pH and oxygen cannot be assumed to be additive. A full factorial analysis of pH and oxygen tension would therefore be required to resolve these interactions, but this was outside the scope of the present short communication.

Taken together, this study reveals a complex landscape of antimicrobial susceptibility caused by the different β-lactamases, in some cases even to a degree where current EUCAST/CLSI breakpoints might lead to treatment failure (or success) in specific anatomical sites. However, this warrants further research. Importantly, the effect of pH and anaerobiosis not only depends on the specific β-lactamase but also on the specific β-lactam. This could have major implications not only for our understanding of the evolution and ecology of antimicrobial resistance and the selective impact of the use of different β-lactam antibiotics, but also for the predictive value of *in vitro* susceptibility testing, which often tests a narrow range of β-lactams and uses the results as proxies to predict general susceptibility to other compounds.

Even though further research is necessary, these findings suggest that, in the future, we should more fully integrate knowledge of the exact antimicrobial resistance gene, the exact compound used, and the specific micro-environmental conditions at the infection or colonization site in research and clinical microbiology.

## Data Availability

All MIC data and the conditions they were generated under are provided in the Data S1.

## SUPPLEMENTAL MATERIAL

Fig. S1 spectrum.01453-26-s0001.docx Effect of pH on the MIC.

Fig. S2 spectrum.01453-26-s0002.docx Effect of anaerobiosis on the MIC.

Fig. S3 spectrum.01453-26-s0003.docx Heatmaps showing the effect of anaerobiosis and pH on the MICs.

Supplemental data spectrum.01453-26-s0004.xlsx All MIC data and the conditions they were generated under.

Supplemental tables spectrum.01453-26-s0005.docx Strains, plasmids, and primers used.

ASM does not own the copyrights to Supplemental Material that may be linked to, or accessed through, an article. The authors have granted ASM a non-exclusive, world-wide license to publish the Supplemental Material files. Please contact the corresponding author directly for reuse.

## References

[1] World Health Organization. 2023. Antimicrobial resistance. Fact sheet. Available from: https://www.who.int/news-room/fact-sheets/detail/antimicrobial-resistance

[2] Njage PMK, van Bunnik B, Munk P, Marques ARP, Aarestrup FM. 2023. Association of health, nutrition, and socioeconomic variables with global antimicrobial resistance: a modelling study. Lancet Planet Health 7:e888–e899. doi:10.1016/S2542-5196(23)00213-9 37940209

[3] Ortiz-Miravalles L, Prieto A, Kieffer N, Vergara E, Cantón R, San Millán Á, Baquero F, Hipólito A, Escudero JA. 2025. Effect of oxygen on antimicrobial resistance genes from a one health perspective. Sci Total Environ 979:179523. doi:10.1016/j.scitotenv.2025.179523 40286623

[4] Anbo M, Otani S, Ivanova M, Nielsen HN, Jensen JD, Svendsen CA, Pang C, Aarestrup FM. 2026. Contrasting pH optima of β-lactamases CTX-M and CMY influence *Escherichia coli* fitness and resistance ecology. Appl Environ Microbiol 92:e0177525. doi:10.1128/aem.01775-25 41457317 PMC12863049

[5] Bush K, Bradford PA. 2019. Interplay between β-lactamases and new β-lactamase inhibitors. Nat Rev Microbiol 17:295–306. doi:10.1038/s41579-019-0159-8 30837684

[6] Krco S, Davis SJ, Joshi P, Wilson LA, Monteiro Pedroso M, Douw A, Schofield CJ, Hugenholtz P, Schenk G, Morris MT. 2023. Structure, function, and evolution of metallo-β-lactamases from the B3 subgroup-emerging targets to combat antibiotic resistance. Front Chem 11:1196073. doi:10.3389/fchem.2023.1196073 37408556 PMC10318434

[7] Bonomo RA, Burd EM, Conly J, Limbago BM, Poirel L, Segre JA, Westblade LF. 2018. Carbapenemase-producing organisms: a global scourge. Clin Infect Dis 66:1290–1297. doi:10.1093/cid/cix893 29165604 PMC5884739

[8] Lemaire S, Van Bambeke F, Mingeot-Leclercq M-P, Glupczynski Y, Tulkens PM. 2007. Role of acidic pH in the susceptibility of intraphagocytic methicillin-resistant *Staphylococcus aureus* strains to meropenem and cloxacillin. Antimicrob Agents Chemother 51:1627–1632. doi:10.1128/AAC.01192-06 17307986 PMC1855560

[9] König C, Simmen HP, Blaser J. 1993. Effect of pathological changes of pH, pO2 and pCO2 on the activity of antimicrobial agents *in vitro*. Eur J Clin Microbiol Infect Dis 12:519–526. doi:10.1007/BF01970957 8404912

[10] König C, Blaser J. 1995. Effect of pO2 and pH on synergy of tazobactam and β-lactam antibiotics against β-lactamase producing enterobacteriaceae. J Antimicrob Chemother 36:513–519. doi:10.1093/jac/36.3.513 8830015

[11] D’Amato RF, Thornsberry C. 1979. Calcium and magnesium in mueller-hinton agar and their influence on disk diffusion susceptibility results. Curr Microbiol 2:135–138. doi:10.1007/BF02605869

[12] Kenny MA, Pollock HM, Minshew BH, Casillas E, Schoenknecht FD. 1980. Cation components of mueller-hinton agar affecting testing of *Pseudomonas aeruginosa* susceptibility to gentamicin. Antimicrob Agents Chemother 17:55–62. doi:10.1128/AAC.17.1.55 6766293 PMC283726

[13] Livermore DM, Corkill JE. 1992. Effects of CO_2_ and pH on inhibition of TEM-1 and other beta-lactamases by penicillanic acid sulfones. Antimicrob Agents Chemother 36:1870–1876. doi:10.1128/AAC.36.9.1870 1329633 PMC192202

[14] Fornara AM, Danel F, Livermore DM. 1997. pH effects on the inhibition of extended-spectrum and classical TEM beta-lactamases by penicillanic acid sulphones. J Antimicrob Chemother 39:89–93. doi:10.1093/jac/39.1.89 9044033

[15] Wilks JC, Slonczewski JL. 2007. pH of the cytoplasm and periplasm of *Escherichia coli*: rapid measurement by green fluorescent protein fluorimetry. J Bacteriol 189:5601–5607. doi:10.1128/JB.00615-07 17545292 PMC1951819

[16] Dixon M, Webb EC. 1979. The enzymes. 3rd edition, p 1116. Academic Press.

[17] Rasia RM, Vila AJ. 2002. Exploring the role and the binding affinity of a second zinc equivalent in *B. cereus* metallo-beta-lactamase. Biochemistry 41:1853–1860. doi:10.1021/bi010933n 11827530

