# Supplementary material for: Environmental factors modulate β-lactamase phenotypes: effect of pH and redox potential on β-lactam resistance
Source: Microbiol Spectr. 2026 Jul 17;14(8):e01453-26. doi: 10.1128/spectrum.01453-26 (PMC13436003; doi:10.1128/spectrum.01453-26)
Supplement: Supplementary file 1 [file spectrum.01453-26-s0001.docx]

**Supplementary figures (Figure S1 panels all genes)**

**Figure S1 Influence of pH on the MIC of different β-lactamases.** A: Effect of media pH on the MIC of the β-lactamases to the β-lactam. Each β-lactamase shows a distinct pH optimum, suggesting that pH can play an integral role in shaping resistomes. Dashed lines indicate the maximum and minimum concentrations that were assayed, while the horizontal red and blue lines indicate resistant and susceptible CLSI M100 Enterobacterales MIC breakpoints, respectively, where available. No CLSI clinical breakpoint was available for temocillin or for the cefotaxime/ceftazidime plus clavulanic acid experimental combinations. B: Heatmap showing the effect of media at pH 5 on MIC where each substrate/gene combination is coloured according to the log2 fold change between the MIC at pH 5 and the MIC at pH 7 (red for increased resistance and blue for increased susceptibility). This was normalized to the corresponding log2 fold change in MIC of the wild-type, to give a species-agnostic estimate. Each condition was tested with 3 replicates.
