# Supplementary material for: Environmental factors modulate β-lactamase phenotypes: effect of pH and redox potential on β-lactam resistance
Source: Microbiol Spectr. 2026 Jul 17;14(8):e01453-26. doi: 10.1128/spectrum.01453-26 (PMC13436003; doi:10.1128/spectrum.01453-26)
Supplement: Supplementary file 2 [file spectrum.01453-26-s0002.docx]

**Supplementary figures (Figure S2 panels all genes)**

Figure S2 Influence of oxygen pressure on the MIC of different β-lactamases at pH 7. A: Effect of anaerobiosis on the MIC of the β-lactamases to the different β-lactams. Dashed lines indicate the maximum and minimum concentrations that were assayed, while the horizontal red and blue lines indicate resistant and susceptible CLSI M100 Enterobacterales MIC breakpoints, respectively, where available. No CLSI clinical breakpoint was available for temocillin or for the cefotaxime/ceftazidime plus clavulanic acid experimental combinations. B: Heatmap showing the effect of anaerobiosis on MIC where each substrate/gene combination is coloured according to the log2 fold change between the anaerobic MIC and the aerobic MIC (red for increased resistance and blue for increased susceptibility). This was normalized to the corresponding log2 fold change in MIC of the wild-type, to give a species-agnostic estimate. Each condition was tested with 3 replicates.
