# Supplementary material for: Environmental factors modulate β-lactamase phenotypes: effect of pH and redox potential on β-lactam resistance
Source: Microbiol Spectr. 2026 Jul 17;14(8):e01453-26. doi: 10.1128/spectrum.01453-26 (PMC13436003; doi:10.1128/spectrum.01453-26)
Supplement: Supplementary file 3 [file spectrum.01453-26-s0003.docx]

**Supplementary figures 3**

Supplemental figure 3.1: Heatmaps showing the effect of pH (grey panel above each heatmap) on the MIC of K12 relative to MICs recorded at pH 7. Each condition was assayed in triplicate and the mean was used to calculate log2 fold changes (L2FC), where an increase in MIC is indicated by red, and a decrease with blue.

Supplemental figure 3.2: The effect of anaerobiosis on the MIC of K12 relative to standard MICs (aerobic) at pH 7. Each condition was assayed in triplicate and the mean was used to calculate log2 fold changes (L2FC), where an increase in MIC is indicated by red, and a decrease with blue.

Supplemental figure 3.3: The effect of pH 6 on the MIC of several β-lactamases relative to MIC performed at pH 7. Each condition was assayed in triplicate and the mean was used to calculate log2 fold changes (L2FC), where an increase in MIC is indicated by red, and a decrease with blue. These changes were normalized to the corresponding log2 fold change in MIC of the wild-type.

Supplemental figure 3.4: The effect of pH 8 on the MIC of several β-lactamases relative to MIC performed at pH 7. Each condition was assayed in triplicate and the mean was used to calculate log2 fold changes (L2FC), where an increase in MIC is indicated by red, and a decrease with blue. These changes were normalized to the corresponding log2 fold change in MIC of the wild-type.
