# Supplementary material for: Environmental factors modulate β-lactamase phenotypes: effect of pH and redox potential on β-lactam resistance
Source: Microbiol Spectr. 2026 Jul 17;14(8):e01453-26. doi: 10.1128/spectrum.01453-26 (PMC13436003; doi:10.1128/spectrum.01453-26)
Supplement: Supplementary file 4 [file spectrum.01453-26-s0005.docx]

**Supplementary tables**

Table S1.	Strains and plasmids used in the study.

| **Name** | **Comment** | **Source** |
| --- | --- | --- |
| *E. coli* K12 MG1655 | Wildtype | Anbo et al. 2025 |
| *E. coli* DH5ɑpir | Plasmid propagation | Anbo et al. 2025 |
| K12 OXA-48 | K12 attTn7::pMA1 OXA-48 | This study |
| K12 OXA-232 | K12 attTn7::pMA1 OXA-232 | This study |
| K12 OXA-181 | K12 attTn7::pMA1 OXA-181 | This study |
| K12 VIM-1 | K12 attTn7::pMA1 VIM-1 | This study |
| K12 VIM-2 | K12 attTn7::pMA1 VIM-2 | This study |
| K12 IMP-1 | K12 attTn7::pMA1 IMP-1 | This study |
| K12 IMP-4 | K12 attTn7::pMA1 IMP-4 | This study |
| K12 NDM-1 | K12 attTn7::pMA1 NDM-1 | This study |
| K12 NDM-5 | K12 attTn7::pMA1 NDM-5 | This study |
| K12 KPC-2 | K12 attTn7::pMA1 KPC-2 | This study |
| K12 KPC-3 | K12 attTn7::pMA1 KPC-3 | This study |
| K12 TEM-1 | K12 attTn7::pMA1 TEM-1 | This study |
| K12 TEM-2 | K12 attTn7::pMA1 TEM-2 | This study |
| K12 TEM-52 | K12 attTn7::pMA1 TEM-52 | This study |
| K12 SHV-12 | K12 attTn7::pMA1 SHV-12 | This study |
| K12 GES-5 | K12 attTn7::pMA1 GES-5 | This study |
| TWIW_02_HRV_ZAG_BL_058 | Source of OXA-48 gene | Nag et al. 2023 |
| TWIW_01_PAK_PES_002 | Source of OXA-232 gene | Nag et al. 2023 |
| MG1655-sfGFP-blaOXA-181 | Source of OXA-181 gene | Qinqin study 2022 (not published) |
| TWIW_01_DEU_MAG_047 | Source of VIM-1 gene | Nag et al. 2023 |
| TWIW_02_NGA_ILE_BN_070 | Source of VIM-2 gene | Nag et al. 2023 |
| TWIW_01_THA_BAN_022 | Source of IMP-1 gene | Nag et al. 2023 |
| DTU_2022_1024639_1_MG_PT_PE_1025 sewage sample **DNA** | Source of IMP-4 gene | Martiny et al. 2025 |
| TWIW_02_NGA_ILE_BN_048 | Source of NDM-1 gene | Nag et al. 2023 |
| MG1655-sfGFP-NDM-5 | Source of NDM-5 gene | Qinqin study 2022 (not published) |
| MG1655-sfGFP-KPC-2 | Source of KPC-2 gene | Qinqin study 2022 (not published) |
| TWIW_01_ITA_MIL_050 | Source of KPC-3 gene | Nag et al. 2023 |
| TWIW_01_PRY_CIU_FMNU_008 | Source of TEM-1 gene | Nag et al. 2023 |
| TWIW_01_FRA_VAL_062 | Source of TEM-2 gene | Nag et al. 2023 |
| 2010-60-1435-1 | Source of TEM-52 gene | Agersø et al. 2012 |
| TWIW_01_PRY_CIU_RH_027 | Source of SHV-12 gene | Nag et al. 2023 |
| TWIW_02_NGA_ILE_BN_052 | Source of GES-5 gene | Nag et al. 2023 |
| Plasmids |  |  |
| pMA1 BG28 | pUC18R6k-mini-tn7T-gm with regulon BG28 BCD2 integrated | Anbo et al 2025 |
| Tn7 helper plasmid | Tn7 Helper plasmid | Addgene plasmid #141161, Jasinska et al. 2020 |
| pMA1 OXA-48 | pMA1 BG28 with OXA-48 integrated | This study |
| pMA1 OXA-232 | pMA1 BG28 with OXA-232 integrated | This study |
| pMA1 OXA-181 | pMA1 BG28 with OXA-181 integrated | This study |
| pMA1 VIM-1 | pMA1 BG28 with VIM-1 integrated | This study |
| pMA1 VIM-2 | pMA1 BG28 with VIM-2 integrated | This study |
| pMA1 IMP-1 | pMA1 BG28 with IMP-1 integrated | This study |
| pMA1 IMP-4 | pMA1 BG28 with IMP-4 integrated | This study |
| pMA1 NDM-1 | pMA1 BG28 with NDM-1 integrated | This study |
| pMA1 NDM-5 | pMA1 BG28 with NDM-5 integrated | This study |
| pMA1 KPC-2 | pMA1 BG28 with KPC-2 integrated | This study |
| pMA1 KPC-3 | pMA1 BG28 with KPC-3 integrated | This study |
| pMA1 TEM-1 | pMA1 BG28 with TEM-1 integrated | This study |
| pMA1 TEM-2 | pMA1 BG28 with TEM-2 integrated | This study |
| pMA1 TEM-52 | pMA1 BG28 with TEM-52 integrated | This study |
| pMA1 SHV-12 | pMA1 BG28 with SHV-12 integrated | This study |
| pMA1 GES-5 | pMA1 BG28 with GES-5 integrated | This study |

Agersø Y, Aarestrup FM, Pedersen K, Seyfarth AM, Struve T, Hasman H. 2012. Prevalence of extended-spectrum cephalosporinase (ESC)-producing *Escherichia coli* in Danish slaughter pigs and retail meat identified by selective enrichment and association with cephalosporin usage. J Antimicrob Chemother 67:582-8.

Anbo M, Otani S, Ivanova M, Nielsen HN, Jensen JD, Svendsen CA, Pang C, Aarestrup FM. 2025. Contrasting pH optima of β-lactamases CTX-M and CMY influence *Escherichia coli* fitness and resistance ecology. Appl Environ Microbiol 92:e0177525.

Jasinska W, Manhart M, Lerner J, Gauthier L, Serohijos AWR, Bershtein S. 2020. Chromosomal barcoding of E. coli populations reveals lineage diversity dynamics at high resolution. Nat Ecol Evol 4:437-452.

Martiny HM, Munk P, Fuschi A, Becsei Á, Pyrounakis N, Brinch C; Global Sewage Consortium; Larsson DGJ, Koopmans M, Remondini D, Csabai I, Aarestrup FM. 2025. Geographics and bacterial networks differently shape the acquired and latent global sewage resistomes. Nat Commun 16:10278.

Nag S, Larsen G, Szarvas J, Birkedahl LEK, Gulyás GM, Ciok WJ, Lagermann TM, Tafaj S, Bradbury S, Collignon P, Daley D, Dougnon V, Fabiyi K, Coulibaly B, Dembélé R, Nikiema G, Magloire N, Ouindgueta IJ, Hossain ZZ, Begum A, Donchev D, Diggle M, Turnbull L, Lévesque S, Berlinger L, Sogaard KK, Guevara PD, Valderrama CD, Maikanti P, Amlerova J, Drevinek P, Tkadlec J, Dilas M, Kaasch A, Westh HT, Bachtarzi MA, Amhis W, Salazar CES, Villacis J, Lúzon MAD, Palau DB,

Duployez C, Paluche M, Asante-Sefa S, Moller M, Ip M, Mareković I, Pál-Sonnevend A, Cocuzza CE, Dambrauskiene A, Macanze A, Cossa A, Mandomando I, Nwajiobi-Princewill P, Okeke IN, Kehinde AO, Adebiyi I, Akintayo I, Popoola O, Onipede A, Blomfeldt A, Nyquist NE, Bocker K, Ussher J, Ali A, Ullah N, Khan H, Gustafson NW, Jarrar I, Al-Hamad A, Luvira V, Paveenkittiporn W, Baran I, Mwansa JCL, Sikakwa L, Yamba K, Hendriksen RS, Aarestrup FM. 2023. Whole genomes from bacteria collected at diagnostic units around the world 2020. Sci Data 10:628.

Table S2.	List of Primers used in this study. Sequences are given in 5’-3’ direction. Underlined sequences are homologous to pMA1 BG28 and are used to add overhangs to genes so they can be integrated into this vector with gibson assembly.

| **Name** | **Sequence** | **Purpose** |
| --- | --- | --- |
| Pr45 BCD2 rev | ATTAGAAAACCTCCTTAGCA | Linearization of pMA1 BG28 |
| Pr69 pMA1 linear fw | AGATTCCAGGCGGTGCCATG | Linearization of pMA1 BG28 |
| Pr9 mini-tn7 seq fw | TATTAAAGAGGGGCGTGGG | Verification of insert in pMA1 BG28 |
| Pr10 mini-tn7 seq rev | AGAGCGTTCACCGACAAA | Verification of insert in pMA1 BG28 |
| Pr54 glmS fw | GGAAGAGGTGATTGCACCGA | Tn7 integration verification |
| Pr19 BCD2 fw | AGGGCCCAAGTTCACTTAAA | Sanger sequencing |
| Pr23 mini-tn7 rev2 | CAAGCTAGAGAGTCATTACC | Sanger sequencing |
| Pr78 OXA-181 gibs fw | ATCTTAATCATGCTAAGGAGGTTTTCTAATGCGTGTATTAGCCTTA | PCR Amplification |
| Pr79 OXA-181 gibs rev | AGGCGAATACCATGGCACCGCCTGGAATCTCTAGGGAATAATTTTCTCCTG | PCR Amplification |
| Pr121 OXA-48 rev | AGGCGAATACCATGGCACCGCCTGGAATCTCTAGGGAATAATTTTTTCCTG | PCR Amplification |
| Pr114 VIM-1 fw | ATCTTAATCATGCTAAGGAGGTTTTCTAATGTTAAAAGTTATTAGTAGT | PCR Amplification |
| Pr115 VIM-1 rev | AGGCGAATACCATGGCACCGCCTGGAATCTCTACTCGGCGACTGAGCG | PCR Amplification |
| Pr116 VIM-2 fw | ATCTTAATCATGCTAAGGAGGTTTTCTAATGTTCAAACTTTTGAGTAAG | PCR Amplification |
| Pr117 VIM-2 rev | AGGCGAATACCATGGCACCGCCTGGAATCTCTACTCAACGACTGAGCG | PCR Amplification |
| Pr118 IMP fw | ATCTTAATCATGCTAAGGAGGTTTTCTAATGAGCAAGTTATCTGTATTC | PCR Amplification |
| Pr119 IMP-1 rev | AGGCGAATACCATGGCACCGCCTGGAATCTTAGTTGCTTGGTTTTGAT | PCR Amplification |
| Pr120 IMP-4 rev | AGGCGAATACCATGGCACCGCCTGGAATCTTAGTTGCTTAGTTTTGATG | PCR Amplification |
| Pr76 NDM-5 gibs fw | ATCTTAATCATGCTAAGGAGGTTTTCTAATGGAATTGCCCAATATTATGC | PCR Amplification |
| Pr77 NDM-5 gibs rev | AGGCGAATACCATGGCACCGCCTGGAATCTCAGCGCAGCTTGTCGG | PCR Amplification |
| Pr74 KPC-2 gibs fw | ATCTTAATCATGCTAAGGAGGTTTTCTAATGTCACTGTATCGCCGTC | PCR Amplification |
| Pr75 KPC-2 gibs rev | AGGCGAATACCATGGCACCGCCTGGAATCTTACTGCCCGTTGACGC | PCR Amplification |
| Pr109 TEM52fw | ATCTTAATCATGCTAAGGAGGTTTTCTAATGAGTATTCAACATTTYC | PCR Amplification |
| Pr110 TEM52rev | AGGCGAATACCATGGCACCGCCTGGAATCTTACCAATGCTTAATCAG | PCR Amplification |
| Pr107 SHV12fw | ATCTTAATCATGCTAAGGAGGTTTTCTAATGCGTTATATTCGCCTGTG | PCR Amplification |
| Pr108 SHV12rev | AGGCGAATACCATGGCACCGCCTGGAATCTTAGCGTTGCCAGTGCTC | PCR Amplification |
| Pr122 GES-5 fw | ATCTTAATCATGCTAAGGAGGTTTTCTAATGCGCTTCATTCACGCACTA | PCR Amplification |
| Pr123 GES-5 rev | AGGCGAATACCATGGCACCGCCTGGAATCTATTTGTCCGTGCTCAGGAT | PCR Amplification |
